# Resilience and work engagement: the mediating role of job crafting behaviours

**DOI:** 10.3389/fpsyg.2026.1826711

**Published:** 2026-10-28

**Authors:** Nelesh Dhanpat

**Affiliations:** Department of Industrial Psychology and People Management, College of Business and Economics, University of Johannesburg, Johannesburg, South Africa

**Keywords:** cognitive crafting, job crafting, mediation, relational crafting, resilience, task crafting, work engagement

## Abstract

**Introduction:**

Despite growing recognition that resilience and work engagement are central to employee wellbeing, the mechanisms through which resilience is associated with engaged work behaviour remain underexplored. Grounded in Conservation of Resources theory, this study examined task, cognitive, and relational job crafting as parallel mediating mechanisms in the resilience-work engagement relationship.

**Methods:**

Cross-sectional data were collected from 332 employees at a large financial services organisation operating across South Africa and Namibia. Resilience, job crafting, and work engagement were assessed using validated measures. A parallel mediation model was tested using PROCESS macro Model 4 with 5,000 bootstrap samples.

**Results:**

Resilience significantly predicted all three crafting behaviours and work engagement. All indirect effects were statistically significant. Cognitive crafting showed the largest estimated indirect effect, followed by relational and task crafting. The model explained 34% of variance in work engagement, with crafting behaviours accounting for approximately 45% of the total effect of resilience on engagement. The direct effect remained significant, indicating partial mediation.

**Discussion:**

Resilient employees actively reshape their work through behavioural, cognitive, and relational crafting, and this proactive activity meaningfully explains how resilience generates engagement. Cognitive reframing of work as purposeful and meaningful emerged as the most potent pathway. Findings carry practical relevance for resilience training, leadership practice, and organisational job design.

## Introduction

Organisations operating in volatile, uncertain, and increasingly demanding work environments (Stofile et al., 2023; Vrontis et al., 2025) have placed growing emphasis on two psychological constructs: resilience and work engagement. Resilience, broadly defined as the capacity to adapt positively in the face of adversity, stress, or significant challenge (Luthans, 2002), has been identified as a key personal resource underpinning employee wellbeing and performance within the organisational psychology literature (Bakker and van Wingerden, 2021; Hartmann et al., 2020). It is foundational to understand how employees react to and cope with adversity in contemporary work contexts (Rook et al., 2023). Work engagement, characterised by vigour, dedication, and absorption in one’s work (Schaufeli et al., 2002), has similarly attracted sustained scholarly interest owing to its well-documented effects on job performance, organisational commitment, and reduced turnover intention (Schneider et al., 2018; Zahari and Kaliannan, 2023). Yet despite the theoretical and practical prominence of both constructs within occupational and organisational psychology, the mechanisms through which resilience relates to engagement remain insufficiently examined in the empirical literature. Within the Job Demands-Resources (JD-R) model, work engagement is conceptualised as the primary positive outcome of adequate job and personal resources (Bakker and Demerouti, 2017), providing a further theoretical rationale for examining resilience, a key personal resource, as a correlate of engagement.

Conceptually, resilience has been understood both as a relatively stable personal trait and as a dynamic process of positive adaptation that can be developed through experience and resource investment (Luthans et al., 2007). The present study adopts this process-oriented view, consistent with its treatment as a personal resource within COR theory. Work engagement, in turn, is most commonly operationalised through its three interrelated dimensions of vigour, dedication, and absorption (Schaufeli et al., 2002), and has been consistently linked to the availability of job and personal resources within the JD-R model (Bakker and Demerouti, 2017). Both constructs have therefore been examined extensively as independent predictors of employee functioning, but considerably less attention has been paid to how they relate to one another, and to the behavioural mechanisms, such as job crafting, through which one may give rise to the other.

A growing body of organisational psychology research has begun to explore the positive relationship between resilience and work engagement (Kašpárková et al., 2018; Ojo et al., 2021; Ibrahim and Hussein, 2024). Resilient employees are better positioned to mobilise and protect their psychological resources (Singh et al., 2023; Bakker and Demerouti, 2017), which may manifest as higher levels of energy, commitment, and absorption at work. These studies have generally found resilience to be a moderate-to-strong predictor of engagement across occupational groups, including healthcare and engineering samples (Ibrahim and Hussein, 2024; Kašpárková et al., 2018), though the mechanisms explaining this association have rarely been tested directly. A small number of studies have examined job crafting explicitly as this mechanism, most notably among healthcare employees in Egypt (Azmi et al., 2025); the present study extends this line of enquiry to a South African and Namibian financial-services setting and disaggregates job crafting into its task, cognitive, and relational forms rather than treating it as a single composite mediator. Understanding these mechanisms is not merely of theoretical interest; it has direct implications for how practitioners and organisations develop, retain, and support their workforces (Rees et al., 2015; Robertson et al., 2015).

Job crafting has emerged as a construct of considerable importance within the organisational psychology (Dhanpat, 2022) and work behaviour literature (Tims et al., 2022) attracting growing scholarly attention across diverse occupational and cultural contexts (Dhanpat, 2025; Lichtenthaler and Fischbach, 2019; Rudolph et al., 2017). In an era characterised by rapid organisational change, increased job demands, and evolving role expectations, employees can no longer rely solely on organisationally prescribed job designs to meet their needs for meaning, autonomy, and growth (Wrzesniewski and Dutton, 2001; Tims et al., 2012). Job crafting offers a bottom-up, employee-driven complement to traditional top-down job design approaches (Roczniewska et al., 2023) enabling individuals to proactively align their work with their personal strengths, values, and motivations (Tims and Akkermans, 2020). Despite its growing prominence internationally, job crafting remains underexplored in the South African organisational context, with a recent review identifying only seven empirical studies conducted between 2010 and 2020 (Dhanpat, 2022), highlighting the need for further investigation in this setting.

One promising yet insufficiently examined mechanism linking resilience to engagement is job crafting, defined as the proactive, self-initiated behaviours through which employees alter the task, cognitive, and relational boundaries of their work (Wrzesniewski and Dutton, 2001; Berg et al., 2013; Tims et al., 2012). Job crafting is an active resource-building strategy (Bai et al., 2021) that reflects employees’ agency in shaping their own work experience (Munnisunker and Dhanpat, 2025; Marx et al., 2025). From a Conservation of Resources (COR) perspective (Hobfoll, 1989), individuals who possess abundant personal resources such as resilience are better positioned and more motivated to engage in resource-seeking and resource-building behaviours at work. Job crafting, as a proactive form of work behaviour (Demerouti, 2026), represents precisely this kind of resource investment activity. The resources accumulated through crafting may in turn fuel greater levels of work engagement, suggesting that job crafting is a theoretically coherent and practically relevant mechanism through which resilience operates. Although resilience, job crafting, and work engagement have each attracted substantial independent attention, the small number of studies that model job crafting as the mechanism linking resilience to engagement do not agree on the direction of this relationship. Some, consistent with the direction tested here, treat resilience as an antecedent of crafting (Azmi et al., 2025); others report the reverse pattern, with job crafting predicting resilience (Xiao et al., 2025; Mandaviya, 2020; Van Wingerden and Poell, 2019), including an analysis applying a different statistical model to the same organisational dataset used in the present study (Munnisunker and Dhanpat, 2025). This mixed evidence reflects the cross-sectional nature of most research in this area, including the present study, and is returned to the Limitations section. The present study addresses the gap in this literature by testing a parallel mediation model in which task, cognitive, and relational crafting simultaneously mediate the resilience-engagement relationship in a Southern African financial-services sample, disaggregating job crafting into its constituent forms so as to identify which specific crafting behaviours are most strongly implicated in this pathway.

### Job crafting: task, cognitive, and relational forms

Job crafting theory has developed across two primary perspectives. Wrzesniewski and Dutton (2001) originally conceptualised job crafting as employee-initiated changes to the task, relational, and cognitive boundaries of work. Tims and Bakker (2010) subsequently reframed job crafting within the job demands-resources (JD-R) model, positioning it as the proactive adjustment of job demands and resources. Zhang and Parker (2019) later proposed a synthesis of these two perspectives, while Bindl et al. (2019) introduced a further distinction between approach-oriented and avoidance-oriented crafting behaviours. The present study adopts Wrzesniewski and Dutton’s (2001) conceptualisation, as operationalised by Slemp and Vella-Brodrick (2013), given its emphasis on the meaning-making and identity dimensions of crafting that are most theoretically relevant to the resilience-engagement pathway examined here.

The concept of job crafting was first proposed by Wrzesniewski and Dutton (2001, p. 179), who defined it as “the physical and cognitive changes individuals make in the task or relational boundaries of their work.” Within the job demands-resources framework, job crafting occurs as employees proactively adjust the resources and demands in their work to align with their personal skills, values, and preferences (Bakker and Demerouti, 2007). These adjustments, whether to tasks, work relationships, or work perceptions, allow employees to shape the meaningfulness of their work and their sense of work identity (Berg et al., 2013), and are associated with a range of positive outcomes including higher work engagement, job satisfaction, thriving, and resilience (Berg et al., 2008; Knight et al., 2019). Despite this established evidence base, a review of the literature identified only seven empirical studies on job crafting in the South African context between 2010 and 2020, indicating a significant gap (Dhanpat, 2022). The present study addresses this gap by examining job crafting using Wrzesniewski and Dutton's (2001) three-form conceptualisation, which distinguishes between task, cognitive, and relational crafting.

Task crafting involves proactive, tangible changes to the nature, scope, or amount of work one performs. Employees may alter which tasks they complete, how they complete them, or when, and may elect to take on additional tasks to increase variety and develop new skills (Lazazzara et al., 2020; Geldenhuys et al., 2021). Berg et al. (2013) noted that task crafting may also involve devoting greater attention to the aspects of work one finds most meaningful, or re-engineering tasks entirely where circumstances do not permit simple task addition. Task crafting is outwardly focused and situationally grounded, representing a behavioural form of control over one’s work aimed at improving motivation and work quality (Hornung, 2019; Devotto and Wechsler, 2019). Within a Conservation of Resources framework (Hobfoll, 1989), task crafting generates resources primarily through mastery experiences and the expansion of role boundaries.

Cognitive crafting, by contrast, is inwardly directed and operates at the psychological level without altering actual job tasks. It involves employees reframing how they perceive and interpret their work to align it more closely with their personal values, purpose, and identity (Wrzesniewski and Dutton, 2001). This may involve viewing one’s work in terms of its broader impact, focusing attention on the aspects of work that carry personal significance, or drawing explicit connections between one’s work and one’s sense of self (Berg et al., 2013). Examples include purposefully focusing on the positive components of work (Vuori et al., 2012) or linking work tasks to areas of genuine personal interest (Batova, 2018). Although Tims and Bakker (2010) characterised cognitive crafting as a form of avoidance, Niessen et al. (2016) repositioned it as a meaningful mechanism for achieving person-job fit through identity and meaning reconstruction. Of the three crafting forms, cognitive crafting generates resources through the enhancement of psychological meaningfulness, which makes it theoretically particularly relevant to engagement outcomes.

Relational crafting involves exercising agency over one’s workplace interactions, including who one interacts with, how frequently, and how deeply (Wrzesniewski and Dutton, 2001). Driven by the fundamental need to connect with others, employees may gravitate towards colleagues who are supportive and affirming, and away from those who are draining or difficult (Geldenhuys et al., 2021). Relational crafting may be expansion-oriented, such as including more colleagues in collaborative work, or contraction-oriented, such as reducing meetings with particular individuals (Rofcanin et al., 2019). Berg et al. (2013) emphasised that relational crafting can also involve extending support and help to others, reframing existing relationships as more purposeful, or investing more deeply in relationships that instil a sense of pride and significance in one’s work. Together with task crafting, relational crafting involves tangible changes to the work context, whereas cognitive crafting operates through intangible perceptual shifts (Lichtenthaler and Fischbach, 2016). Relational crafting generates resources through social connection, support, and a strengthened sense of belonging.

Taken together, the three forms of job crafting represent distinct but complementary resource-building mechanisms. Because each operates through a different pathway, their individual contributions to the resilience-work engagement relationship are unlikely to be equivalent, which makes it necessary to examine them as parallel mediators rather than as a composite whole.

The primary contribution of the present study is its disaggregation of job crafting into task, cognitive, and relational forms within a single parallel mediation model, allowing the relative importance of each crafting pathway between resilience and engagement to be identified rather than assumed. This extends prior single-mediator tests of the resilience-crafting-engagement chain, including recent work in the Egyptian healthcare sector (Azmi et al., 2025), to a Southern African financial-services context in which job crafting research remains scarce. In doing so, the study also extends COR theory by treating job crafting as a resource-building mechanism linking resilience to engagement, and addresses the limited empirical attention given to resilience as an antecedent of job crafting behaviour.

## Hypotheses

Based on the theoretical framework and empirical evidence reviewed above, the following hypotheses are proposed.

*Hypothesis 1*: Resilience will be positively and significantly associated with work engagement.

*Hypothesis 2*: Resilience will be positively and significantly associated with each of the three job crafting behaviours (task, cognitive, and relational crafting).

*Hypothesis 3*: Each of the three job crafting behaviours will be positively and significantly associated with work engagement.

*Hypothesis 4*: The three job crafting behaviours will collectively and individually mediate the relationship between resilience and work engagement, such that resilience will predict higher levels of job crafting, which in turn will predict higher levels of work engagement.

## Materials and methods

### Sample and procedure

Data were collected from employees at a large Southern African financial services organisation with offices in the Western Cape and Gauteng, South Africa, and in Windhoek, Namibia. The questionnaire was administered electronically via Google Forms, distributed by email to all target population members on 1 November 2023. The data collection period ran for 14 weeks. Hard-copy versions, identical in content to the online survey, were made available to participants who preferred this format. Follow-up reminders were sent at regular intervals to maintain response rates. A convenience sampling strategy was employed, consistent with prior organisational research conducted within single-institution settings (Etikan et al., 2016). *A priori* power analysis using G*Power (Faul et al., 2007) indicated that a sample of 332 was sufficient to detect small-to-medium indirect effects in a parallel mediation model with three mediators at 80% power and *α* = 0.05, consistent with recommendations for bootstrap mediation analysis (Fritz and MacKinnon, 2008). A total of 332 employees completed the survey (*N* = 332). Participation was voluntary and anonymous, and all participants provided informed consent prior to completing the survey. The sample comprised 332 employees (female: 56.93%; male: 43.07%), predominantly aged between 30 and 49 years (69.19%). Most participants identified as Black African (63.55%), were permanently employed (93.37%), held a postgraduate or degree-level qualification (59.94%), and reported more than 10 years of work experience (55.72%). Job levels ranged from entry level to executive, with the largest proportions at entry level (38.25%) and middle management (25.00%).

### Ethical considerations

Ethical approval was obtained from the University of Johannesburg Ethics Committee (clearance code: IPPM-2023-734(M)), and institutional permission to access employees was granted in writing by the organisation. Participation was voluntary and anonymous, and all participants provided informed consent prior to completing the survey. Participants were advised of their right to withdraw at any time without consequence. Data were stored securely on a password-protected device for 5 years, accessible only to the researcher and supervisor.

### Measures

#### Resilience

Resilience was assessed using the Brief Resilience Scale (BRS; Smith et al., 2008), a six-item measure of individuals’ capacity to recover from stressful events. Items are rated on a 5-point scale ranging from 1 (strongly disagree) to 5 (strongly agree), with reverse coding applied to alternate items. A sample item is “I tend to bounce back quickly after hard times.”. Previous studies have noted an acceptable Cronbach’s alpha coefficient of 0.83 and 0.90 (Supervía et al., 2022). Factor analysis supported the use of a composite score for the BRS in the present sample (KMO = 0.69; Bartlett’s test: *χ*^2^ = 466.57, df = 15, *p* < 0.001); full details are reported in Munnisunker and Dhanpat (2025). Although two components with eigenvalues greater than 1 were extracted, the loading pattern supported a unidimensional interpretation consistent with the original scale construction (Smith et al., 2008); a composite score was therefore used.

#### Job crafting

Job crafting was assessed using the Job Crafting Questionnaire (JCQ; Slemp and Vella-Brodrick, 2013), a 15-item scale with three subscales measuring task, cognitive, and relational crafting. Items are rated on a 6-point scale ranging from 1 (hardly ever) to 6 (very often). A sample item is “Choose to take on additional tasks at work” (task crafting). The three-factor structure of the JCQ was confirmed in the present sample (KMO = 0.88; Bartlett’s test: *χ*^2^ = 1824.28, df = 105, *p* < 0.001; cumulative variance = 57.22%); full factor analytic details are reported in Munnisunker and Dhanpat (2025). Acceptable reliability for the three subscales (*α* between 0.73 and 0.88) and the overall scale (*α* > 0.70) has been demonstrated in prior research (Mkhwanazi and Dhanpat, 2023).

#### Work engagement

Work engagement was assessed using the nine-item Utrecht Work Engagement Scale (UWES-9; Schaufeli et al., 2006), comprising three subscales measuring vigour, dedication, and absorption. Items are rated on a 7-point scale ranging from 0 (never) to 6 (always). A sample item is “At my work, I feel bursting with energy.” The UWES-9 has demonstrated strong internal consistency in prior research, with overall scale reliability coefficients of 0.90 (Carmona-Halty et al., 2019) and 0.91 (Mkhwanazi and Dhanpat, 2023). The unidimensional factor structure of the UWES-9 was confirmed in the present sample (KMO = 0.91; Bartlett’s test: *χ*^2^ = 2001.22, df = 36, *p* < 0.001; cumulative variance = 61.92%); full details are reported in Dhanpat et al. (2019).

### Data analysis

Descriptive statistics and Pearson correlations were computed for all study variables. Internal consistency reliability was assessed using Cronbach’s alpha, with values of 0.70 or above considered acceptable (Pallant, 2020). Prior to the mediation analysis, the assumptions of linearity, normality, and homoscedasticity were examined through inspection of residual plots and the Shapiro–Wilk test; all assumptions were satisfactorily met. The hypothesised parallel mediation model was tested using PROCESS macro-Version 5.0 for SPSS (Hayes, 2022), Model 4, with resilience as the predictor, work engagement as the outcome, and task, cognitive, and relational crafting as simultaneous mediators. Statistical inference for indirect effects was based on 5,000 bootstrap samples with 95% bias-corrected confidence intervals; an indirect effect was considered significant when its confidence interval excluded zero.

## Results

### Preliminary analyses

Prior to testing the hypothesised mediation model, preliminary analyses were conducted to examine descriptive statistics, internal consistency, and bivariate correlations among all study variables. All scales demonstrated acceptable to excellent reliability (Cronbach’s α ranging from 0.71 to 0.92). Means, standard deviations, reliability coefficients, and intercorrelations are presented in Table 1. Resilience was significantly and positively associated with all three job crafting behaviours, task crafting (*r* = 0.25, *p* < 0.001), cognitive crafting (*r* = 0.28, *p* < 0.001), and relational crafting (*r* = 0.23, *p* < 0.001), as well as with work engagement (*r* = 0.36, *p* < 0.001), providing preliminary support for Hypotheses 1 and 2. Work engagement was also significantly correlated with task crafting (*r* = 0.36, *p* < 0.001), cognitive crafting (*r* = 0.49, *p* < 0.001), and relational crafting (*r* = 0.44, *p* < 0.001), supporting Hypothesis 3.

**Table 1:** Means, standard deviations, reliability coefficients, and intercorrelations among study variables.

Variable	*M*	SD	*α*	1	2	3	4	5
1. Resilience	21.93	4.05	0.76	—				
2. Task Crafting	21.88	4.94	0.71	0.25**	—			
3. Cognitive Crafting	23.71	4.48	0.85	0.28**	0.42**	—		
4. Relational Crafting	21.56	4.90	0.80	0.23**	0.26**	0.53**	—	
5. Work Engagement	38.65	9.67	0.92	0.36**	0.36**	0.49**	0.44**	—

*N* = 332. *α* = Cronbach’s alpha. **p < 0.01.

### Mediation analysis

To test Hypothesis 4, a parallel mediation model was estimated using PROCESS macro Model 4 (Hayes, 2022), with resilience as the predictor (*X*), task crafting, cognitive crafting, and relational crafting as the three parallel mediators (*M*1–3), and work engagement as the outcome (*Y*). The overall model was significant and explained 34% of variance in work engagement (*R*^2^ = 0.34, *F*(4, 327) = 42.65, *p* < 0.001).

The total effect of resilience on work engagement was positive and significant [*b* = 0.85, SE = 0.12, *t* = 6.89, *p* < 0.001, 95% CI (0.60, 1.09)], supporting Hypothesis 1. Resilience was a significant positive predictor of all three job crafting behaviours. Specifically, resilience significantly predicted task crafting (*b* = 0.30, SE = 0.07, *t* = 4.65, *p* < 0.001), cognitive crafting (*b* = 0.31, SE = 0.06, *t* = 5.36, *p* < 0.001), and relational crafting (*b* = 0.28, SE = 0.06, *t* = 4.27, *p* < 0.001), supporting Hypothesis 2. All three crafting behaviours were also significant positive predictors of work engagement in the mediation model: task crafting (*b* = 0.29, SE = 0.10, *t* = 2.94, *p* < 0.01), cognitive crafting (*b* = 0.55, SE = 0.12, *t* = 4.48, *p* < 0.001), and relational crafting (*b* = 0.44, SE = 0.10, *t* = 4.17, *p* < 0.001), supporting Hypothesis 3.

The direct effect of resilience on work engagement, after controlling for all three mediators, remained significant [*b* = 0.47, SE = 0.11, *t* = 4.12, *p* < 0.001, 95% CI (0.24, 0.69)], indicating partial rather than full mediation.

Regarding the indirect effects, the total indirect effect of resilience on work engagement through all three crafting behaviours collectively was significant [*b* = 0.38, SE = 0.08, 95% CI (0.23, 0.56)], as the confidence interval excluded zero. Examining specific indirect effects, cognitive crafting showed the largest estimated indirect effect [*b* = 0.17, SE = 0.06, 95% CI (0.07, 0.30)], followed by relational crafting [*b* = 0.12, SE = 0.04, 95% CI (0.05, 0.22)] and task crafting [*b* = 0.09, SE = 0.04, 95% CI (0.02, 0.17)]. All three specific indirect effects were statistically significant. CollectivelAll three specific indirect effects were statistically significant. To formally test whether these specific indirect effects differed from one another, pairwise bootstrap contrasts were conducted. None of the three contrasts were statistically significant: task versus cognitive crafting [contrast = −0.11, BootSE = 0.07, 95% CI (−0.26, 0.02)], task versus relational crafting [contrast = −0.06, BootSE = 0.06, 95% CI (−0.19, 0.05)], and cognitive versus relational crafting [contrast = 0.05, BootSE = 0.08, 95% CI (−0.11, 0.22)]. Although cognitive crafting produced the numerically largest point estimate, it was not statistically distinguishable from relational or task crafting; this ordering should therefore be interpreted as a descriptive comparison of effect sizes rather than a statistically confirmed difference. Collectively, the indirect pathways accounted for approximately 45% of the total effect of resilience on work engagement, providing support for Hypothesis 4. Full results are reported in Table 2, and the path model is presented in Figure 1.

**Table 2:** Results of parallel mediation analysis: job crafting behaviours as mediators of the resilience–work engagement relationship.

Effect	*b*	SE	*t*	*p*	LLCI	ULCI
Total effect (c path): resilience → engagement	0.85	0.12	6.89	< 0.001	0.60	1.09
Direct Effect (c’ path): resilience → engagement	0.47	0.11	4.12	< 0.001	0.24	0.69
a Paths: resilience → job crafting behaviours						
Resilience → task crafting	0.30	0.07	4.65	< 0.001	0.17	0.43
Resilience → cognitive crafting	0.31	0.06	5.36	< 0.001	0.20	0.43
Resilience → relational crafting	0.28	0.06	4.27	< 0.001	0.15	0.40
b Paths: job crafting behaviours → engagement						
Task crafting → work engagement	0.29	0.10	2.94	< 0.01	0.10	0.48
Cognitive crafting → work engagement	0.55	0.12	4.48	< 0.001	0.31	0.79
Relational crafting → work engagement	0.44	0.10	4.17	< 0.001	0.23	0.64
*Indirect effects (Bootstrap 95% CI)*	*b*	*BootSE*	—	—	*LLCI*	*ULCI*
Total indirect effect	0.38	0.08	—	—	0.23	0.56
Via task crafting	0.09	0.04	—	—	0.02	0.17
Via cognitive crafting	0.17	0.06	—	—	0.07	0.30
Via relational crafting	0.12	0.04	—	—	0.05	0.22

*N* = 332. Mediation analysis conducted using PROCESS Macro Model 4 (Hayes, 2022) with 5,000 bootstrap samples. 95% bias-corrected bootstrap confidence intervals reported for indirect effects. LLCI = lower limit confidence interval; ULCI = upper limit confidence interval. Dashes indicate bootstrap confidence intervals used in place of t and p values for indirect effects. *R*^2^ = 0.34 for the outcome model.

**Figure 1:**
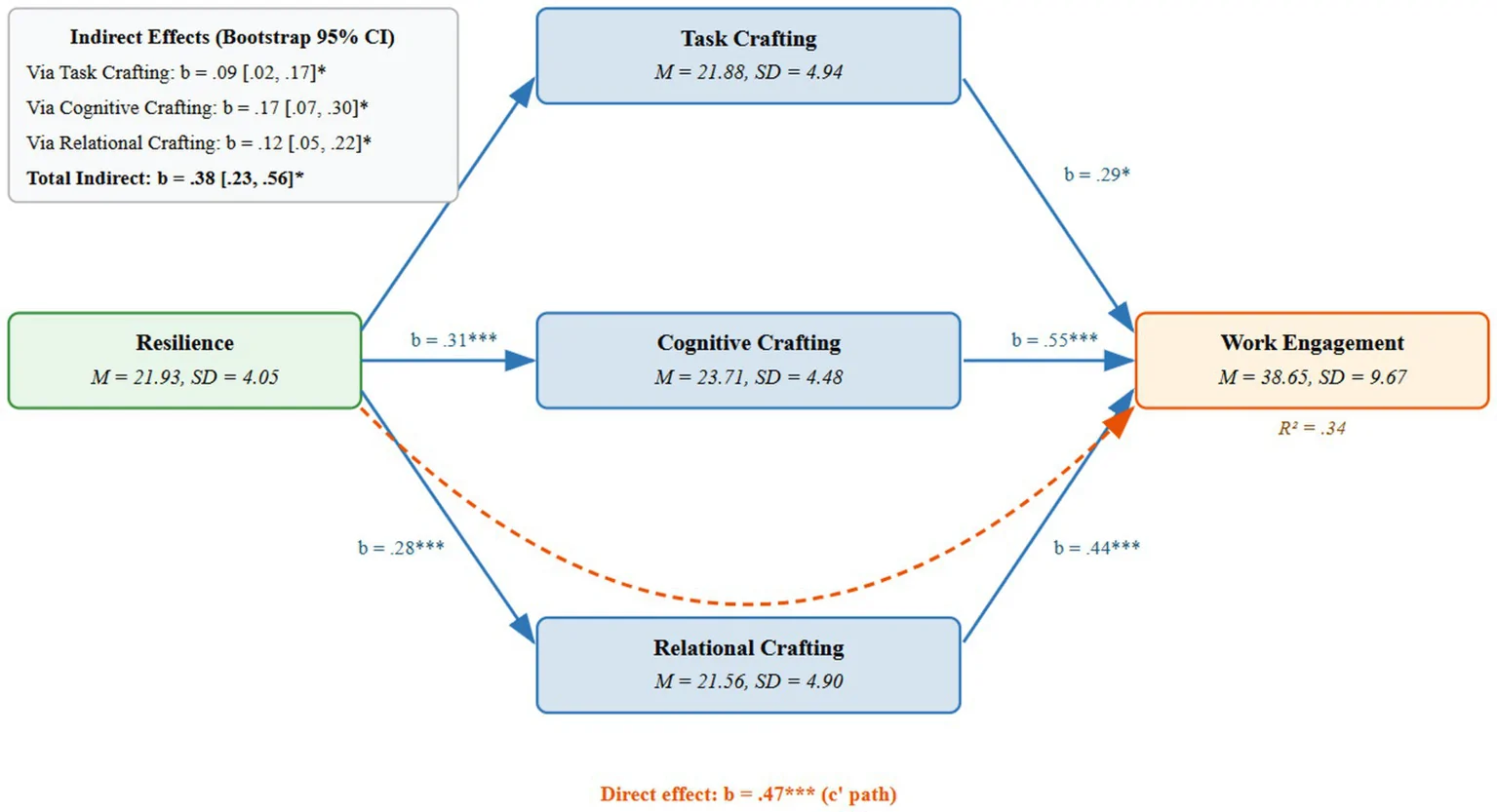
Parallel mediation model: job crafting behaviours as mediators of the resilience–work engagement relationship.

## Discussion

The present study examined whether job crafting behaviours mediate the relationship between resilience and work engagement among a sample of 332 employees. Drawing on Conservation of Resources theory (Hobfoll, 1989), a parallel mediation model was tested in which task, cognitive, and relational crafting were proposed as simultaneous mediating mechanisms. The findings provide strong support for the hypothesised model. Resilience was significantly associated with work engagement both directly and indirectly through all three forms of job crafting, with cognitive crafting showing the largest estimated indirect effect. These findings make several theoretical and practical contributions.

### Resilience and job crafting

Consistent with Hypothesis 2, resilience was a significant positive predictor of all three job crafting behaviours. This finding is theoretically coherent within the COR framework: resilient individuals, by virtue of their rich personal resource reservoir, are better positioned to invest resources proactively (Bardoel and Drago, 2021; Malik, 2023). The positive relationship between resilience and task crafting suggests that resilient employees are more likely to actively seek out challenging or stimulating tasks, expand their role boundaries, and reduce work demands that drain rather than energise them. The relationships with cognitive and relational crafting further indicate that resilience enables employees to reframe their work as more meaningful and to cultivate positive work relationships through two forms of crafting that require psychological confidence and security.

These findings extend prior research on resilience as a personal resource (Luthans et al., 2007; Kuntz et al., 2017; Hartmann et al., 2020) by demonstrating that one of the ways resilience manifests behaviourally at work is through active job crafting. Rather than passively adapting to work conditions, resilient employees appear to actively shape their environments (De Clercq and Pereira, 2019), a finding consistent with the proactive personality literature (Bateman and Crant, 1993; Crant et al., 2016) and with recent calls to understand resilience not merely as a coping response but as a proactive orientation (Butler et al., 2021; Dhanpat et al., 2023).

### Job crafting as a pathway to engagement

All three job crafting behaviours were significant positive predictors of work engagement in the mediation model, consistent with Hypothesis 3 and with the broader job crafting literature (Peral and Geldenhuys, 2016; Mkhwanazi and Dhanpat, 2023). Task crafting, by increasing stimulating job demands or reducing depleting ones, is likely to generate mastery experiences and a sense of competence that underpin the vigour and dedication components of engagement. Relational crafting, through the cultivation of positive work relationships and social support, provides the interpersonal resources that sustain engagement over time. These results constitute a necessary precondition for establishing mediation. Full details of the direct relationship between job crafting and work engagement are reported in Munnisunker and Dhanpat (2025).

Cognitive crafting showed the largest estimated indirect effect within the resilience–engagement relationship. This finding is noteworthy. While task and relational crafting involve observable changes to work behaviour, cognitive crafting operates at the level of meaning-making (Geldenhuys et al., 2021). Resilient individuals who cognitively reframe their work as purposeful, valuable, and connected to broader goals appear to derive particularly high levels of engagement from this process (Morales-Solis et al., 2023) This accords with self-determination theory’s emphasis on psychological meaning as a fundamental driver of intrinsic motivation and engagement (Ryan and Deci, 2000), and with research demonstrating that perceptions of work meaningfulness are among the strongest predictors of engagement (Bakker and Demerouti, 2017; Meng et al., 2022). This pattern may also reflect characteristics of the present sample: most participants held degree-level qualifications and occupied professional or client-facing roles within a financial-services environment, a context in which the meaning and purpose attached to one’s work may be more readily reframed than in roles offering less discretion over how tasks are interpreted. Future research should examine whether the relative strength of cognitive crafting as a mediator generalises to occupational contexts with less scope for interpretive autonomy.

### Partial mediation and the direct effect

Notably, the direct effect of resilience on work engagement remained significant after controlling for the three crafting mediators, indicating partial rather than full mediation. This suggests that job crafting accounts for a meaningful but not complete portion of the resilience–engagement relationship. Other mechanisms not captured in this model are likely to contribute to this relationship. Possible additional pathways include positive emotions generated by resilience (Fredrickson, 2001), self-efficacy beliefs (Bandura, 2013), psychological capital (Luthans et al., 2007), and direct engagement-promoting aspects of resilience such as emotional regulation and positive coping. Future research should investigate these complementary mechanisms to develop a more complete account of how resilience relates to engagement.

### Theoretical implications

This study makes several contributions to the theoretical literature. First, it extends COR theory (Hobfoll, 1989) by providing empirical evidence for the resource caravan mechanism in the context of resilience, job crafting, and engagement. Resilience, as a personal resource, was shown to activate proactive resource-building behaviours (crafting), which in turn fuel engagement—precisely the kind of resource gain spiral that COR theory predicts. Second, the study contributes to the job crafting literature by identifying resilience as a theoretically grounded personal resource antecedent of crafting behaviour. While prior research has examined personality traits and job demands as predictors of crafting (Tims et al., 2012), resilience has received little attention in this role. Third, by disaggregating crafting into its three constituent forms and testing them as parallel mediators, the study reveals differential pathways and highlights the particular importance of cognitive crafting as a meaning-making mechanism in the resilience–engagement relationship.

### Practical implications

The findings carry several practical implications for organisations and human resource practitioners. First, given that resilience predicts both job crafting and work engagement, organisations should invest in resilience development programmes as a means of fostering proactive work behaviour and sustained engagement. Resilience training interventions have demonstrated effectiveness in increasing psychological capital resources (Loghman et al., 2025) and the present findings suggest that such investments may yield benefits for engagement through the activation of crafting behaviours.

Second, the finding that cognitive crafting showed the largest estimated indirect effect suggests that interventions focused on meaning-making at work may be particularly powerful. In practice, this could include manager-led conversations that explicitly connect individual tasks to client or organisational outcomes, onboarding and team-briefing content that clarifies how a role contributes to broader goals, and periodic reflective check-ins in which employees are prompted to identify the aspects of their work they find most meaningful. This emphasis on cognitive reframing should complement rather than substitute for organisational responsibility: encouraging employees to reinterpret their work as meaningful should not be used in place of addressing unreasonable workloads, limited autonomy, or poor psychological safety, which remain the organisation’s responsibility to manage. Third, organisations should consider structural conditions that enable rather than constrain job crafting, including role flexibility, autonomy, and psychological safety, recognising that employees who possess the resilience to craft but lack the environmental conditions to do so may not fully benefit from their personal resources.

## Limitations and future directions

Several limitations of the present study should be acknowledged. First, the cross-sectional design precludes causal inference, and the directionality assumed here (resilience → crafting → engagement) is not the only pattern reported in the literature. Several studies report the reverse pathway, in which job crafting predicts resilience (Xiao et al., 2025; Mandaviya, 2020; van Wingerden and Poell, 2019), including an analysis applying a different statistical model to the same organisational dataset used in the present study (Munnisunker and Dhanpat, 2025). Rather than being contradictory, this pattern is consistent with the possibility of a reciprocal or gain-spiral relationship between resilience and job crafting over time, as anticipated by COR theory (Hobfoll, 2011), in which each resource reinforces the other. The present cross-sectional design cannot adjudicate between these possibilities, and longitudinal or experimental designs are needed to establish directionality and rule out reverse or reciprocal causation. Future research should consider diary study designs, which would allow the dynamic unfolding and fluctuation of crafting behaviours and their relationship with engagement to be examined over time.

The study was conducted in a single occupational context, which may limit the generalisability of findings. Future research should replicate the model across diverse industries, sectors, and cultural contexts to test the boundary conditions of the proposed relationships. In particular, the relative strength of the three crafting pathways may vary depending on occupational characteristics such as task interdependence, role autonomy, and the extent to which cognitive reframing is valued or supported.

An additional limitation concerns sample-level reporting. Demographic variables (gender, age, educational level, and tenure) were not entered as covariates in the mediation model, so their potential confounding influence on the resilience-crafting-engagement relationships cannot be ruled out; future studies should examine whether these pathways hold after controlling for such variables. Relatedly, because the survey was distributed by email to the target population without a fixed, trackable list of recipients, the exact number of employees invited and the resulting response rate could not be determined; this limits the extent to which non-response bias can be evaluated, and future studies should record these figures prospectively.

A further limitation concerns measurement. Resilience, job crafting, and work engagement were each modelled as composite (mean) scores within the PROCESS macro, an approach that does not correct for measurement error in the way a latent-variable structural equation model would. While internal consistency reliability was acceptable for all measures (Cronbach’s alphas ranging from 0.71 to 0.92), standardised factor loadings, composite reliability, average variance extracted, and discriminant validity indices were not computed and are not reported here. Future research should confirm the factor structure and psychometric adequacy of these measures using confirmatory factor analysis before drawing firm conclusions about the relationships reported.

Finally, the present model focused on job crafting as the sole mediating mechanism. Future research should test more complex models that include competing or complementary mediators, such as psychological capital, positive affect, or perceived organisational support, to develop a more comprehensive understanding of the resilience–engagement pathway.

## Conclusion

This study provides the first direct test of job crafting behaviours as mediators of the resilience–work engagement relationship. Drawing on Conservation of Resources theory, the findings demonstrate that resilient employees are more likely to proactively craft their work behaviourally, cognitively, and relationally, and that this crafting activity is a meaningful pathway through which resilience fosters work engagement. Cognitive crafting showed the largest estimated indirect effect, highlighting, highlighting the importance of meaning-making as a mechanism linking personal resources to engaged work behaviour. These findings enrich our understanding of how resilience operates in work contexts and offer actionable insights for practitioners seeking to build engaged and resilient workforces.

## Data Availability

The datasets presented in this article are not readily available because data is confidential and restrictions from the organisation. Requests to access the datasets should be directed to Neleshd@uj.ac.za.
